# Risk factors leading to trabeculectomy surgery of glaucoma patient using Japanese nationwide administrative claims data: a retrospective non-interventional cohort study

**DOI:** 10.1186/s12886-021-01897-4

**Published:** 2021-03-29

**Authors:** Chikako Shirai, Satoru Tsuda, Kunio Tarasawa, Kiyohide Fushimi, Kenji Fujimori, Toru Nakazawa

**Affiliations:** 1 Department of Ophthalmology, Tohoku University Graduate School of Medicine, 1-1 Seiryo-machi, Aoba-ku, Sendai, Miyagi 980-8574 Japan; 2 Department of Health Administration and Policy, Tohoku University Graduate School of Medicine, 1-1 Seiryo-machi, Aoba-ku, Sendai, Miyagi 980-8574 Japan; 3 Department of Health Policy and Informatics, Tokyo Medical and Dental University Graduate School of Medicine, Tokyo, Japan

**Keywords:** Glaucoma, Comorbidity, Concomitant, Administrative claims data, Diagnosis procedure combination (DPC)

## Abstract

**Background:**

Early recognition and management of baseline risk factors may play an important role in reducing glaucoma surgery burdens. However, no studies have investigated them using real-world data in Japan or other countries. This study aimed to clarify the risk factors leading to trabeculectomy surgery, which is the most common procedure of glaucoma surgery, of glaucoma patient using the Japanese nationwide administrative claims data associated with the diagnosis procedure combination (DPC) system.

**Methods:**

It was a retrospective, non-interventional cohort study. Data were collected from patients who were admitted to DPC participating hospitals, nationwide acute care hospitals and were diagnosed with glaucoma between 2012 to 2018. The primary outcome was the risk factors associated with trabeculectomy surgery. The association between baseline characteristics and trabeculectomy surgery was identified using multivariable logistic regression analysis by comparing patients with and without trabeculectomy surgery. Meanwhile, the secondary outcomes included the rate of comorbidities, the rate of concomitant drug use and the treatment patterns of glaucoma eye drops at the index admission. Among patients with trabeculectomy surgery, the risk factors leading to cataract surgery were also evaluated as subgroup analysis.

**Results:**

A total of 29,599 patients included in the analysis, 12,038 and 17,561 patients were in the glaucoma surgery and non-glaucoma surgery cohorts, respectively. The factors associated with the increase in trabeculectomy surgery were having allergies, taking concomitant drugs including cancer, depression, ischemic heart disease and peptic ulcer, being diagnosed with primary open-angle glaucoma and longer length of stay in hospital. In contrast, the factors associated with the decrease in trabeculectomy surgery were having hypertension, taking hypertension drug, age ≥ 80 and female.

**Conclusions:**

Special focus on Japanese patients with glaucoma who have allergy-related comorbidities or take immune, nervous, circulatory or gastrointestinal system-related concomitant drugs seems to be desirable.

**Supplementary Information:**

The online version contains supplementary material available at 10.1186/s12886-021-01897-4.

## Background

Glaucoma is a chronic progressive optic neuropathy that can lead to irreversible blindness, affecting over 70 million adults worldwide. Intraocular pressure (IOP)-lowering therapy is the only effective strategy recognized to date [1–5].

In Japan, glaucoma is the most common cause of blindness, accounting for 28.6% of all blind regression [6]. It has an estimated prevalence of 5% in those aged over 40 years, that is, four million glaucoma patients [7]. Owing to the aging Japanese population, and glaucoma increases with age [8, 9], the future clinical and economic burden to the healthcare system is expected to increase.

Current therapies used to lower IOP are drug treatment (usually eye drops), laser treatment, surgical treatment, or a combination of these treatments are used [5]. Surgical treatment is considered the final step in lowering IOP due to the improvement of drug treatment, and thus, patients with glaucoma undergoing surgical treatment represent severe or drug treatment resistance. Nevertheless, glaucoma surgery has incrementally advanced over the years. The search for safer and less invasive surgeries has been continued with emphasis on newer devices and techniques that use small incisions, a category described as micro-invasive glaucoma surgery [10–12]. New procedures for surgical treatment, however, have their own risks and complications, some of which might be unknown until long-term data become available.

Besides, to the best of our knowledge, there have been no published study results analyzing what background of patients with glaucoma who are receiving surgical treatment in routine clinical practice. Therefore, such study, in particular an analysis of the glaucoma treatment using a large-scale administrative database, resulted in an important and useful information that reflects the current status of treatment and identifies issues to be considered.

The aim of this study was to clarify the risk factors leading to trabeculectomy surgery of glaucoma patients using the Japanese nationwide administrative claims data associated with the diagnosis procedure combination (DPC) system [13, 14]. In addition, the rate of comorbidities, the rate of concomitant drug use, and the treatment patterns of glaucoma eye drops at the index admission in patients with and without trabeculectomy surgery were also explored.

## Methods

### Study design

This was a retrospective non-interventional cohort study using 6-year data (between 1 April 2012 and 31 March 2018) from the Japanese nationwide administrative claims data associated with the DPC system to identify the risk factors leading to trabeculectomy surgery of patients with glaucoma in Japan. Moreover, because glaucoma and cataract are leading causes of blindness worldwide and their co-existence is common in elderly people, the subgroup analysis was performed in patients with combined trabeculectomy and cataract surgery.

This study was registered with UMIN Clinical Trials (UMIN000037878). It was approved by the research ethics committee of the Tohoku University Graduate School of Medicine, Japan (No. 2019–1-897) and conducted in accordance with The Code of Ethics of the World Medical Association (Declaration of Helsinki) for experiments involving human. Because this study was based on secondary analysis of DPC data that had already been anonymized unlinkable, written informed consent from patients was not required.

### Data source and study cohort

The DPC is a national administrative database of a case-mix classification system for acute inpatient care developed in Japan. Details of the system have been described elsewhere [15, 16]. The system was launched in 2003 among 82 special functional hospitals, with a rapidly increasing number of hospitals having adopted the system, which recently includes approximately 7 million inpatients per year from more than 1000 hospitals, covering approximately 90% of all hospitalization to acute care hospital in Japan. The DPC data include administrative claims data and some clinical data. The DPC database includes data on the following elements: patient demographics (e.g., age and gender); primary diagnosis; comorbidities at admission; complications after admission; procedures including surgery, medication and devices used during hospitalization; length of stay; discharge status; and medical expenses [17–19]. The primary diagnosis is limited to one. In order to optimize the accuracy of the recorded diagnoses, the responsible physicians are required to record the diagnosed disease name in the medical charts. A wide variety of centers, including academic, urban and rural hospitals, use the DPC system [20]. These data were coded in the 10th revision of the International Statistical Classification of Disease (ICD10) and also the original Japanese code determined by the Ministry of Health, Labor and Welfare of Japan.

**Fig. 1:**
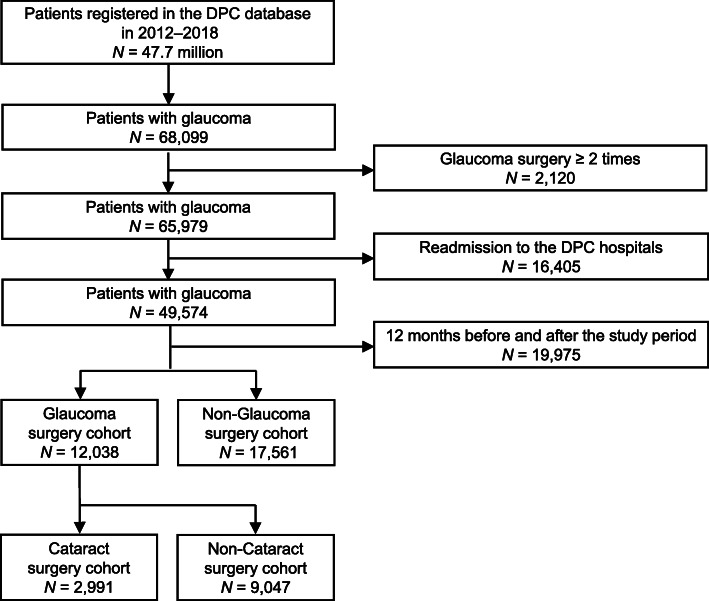
Flowchart for data extraction

For this study, patients were extracted from the DPC data using SQL Server 2014 Management Studio (Microsoft Corporation). Eligible patients were those who were admitted to DPC participating hospitals, that is, nationwide acute care hospitals in Japan and diagnosed with glaucoma. Patients with glaucoma as the disease associated with the code of “highest medical costs” in the DPC database, it means that the most causative disease of hospitalization, were identified based on the following ICD10codes: H401 (normal tension glaucoma, primary open-angle glaucoma and open-angle glaucoma) and H409 (unspecified glaucoma) from 1 April 2012 to 31 March 2018 (study period). The less common types including angle closer glaucoma (H402), secondary glaucoma (H403-H407) and other glaucoma (H408) were excluded. Figure 1 shows the patient selection process. Trabeculectomy surgery was defined as the following claim code for medical procedure: 150335910 (trabeculectomy, TLE). The trabeculotomy (TLO), as well as MIGS and implants, is often additionally used during cataract surgery, and is unlikely to be an indicator of severe glaucoma, while TLE is a standard surgery for glaucoma in the world. More effective in lowering IOP than TLO and rarely used for an additional surgery. To obtain an appropriate outcome of this study, we selected only TLE among glaucoma surgery (GS). The patients with glaucoma who had ≥2 records of GS, readmission to the DPC hospitals, < 12 months post-index continuous enrollment, in-hospital death or missing data during the study period were excluded, and furthermore, this study used the strict inclusion criteria that excluded data for the first and last year during the study period. The patients with only 1 record of TLE from 1 April 2013 to 31 March 2017 was defined as the newly undergone TLE (patients with TLE [GS cohort]).

On the other hand, patients who had not undergone TLE and all other GS (patients without GS [non-GS cohort]) were defined as those who had no record of GS during the study period and who had no record of GS from 1 April 2013 to 31 March 2017. Furthermore, to maintain the independence of observation and to exclude readmission to the DPC hospital, only the data associated with the last admission (index admission) from 1 April 2013 to 31 March 2017 were used as the data of the patients without GS.

Among the patients with TLE, those who have undergone cataract surgery (CS) were defined as patients with CS (CS cohort). CS was defined as the following claim code for medical procedure: 150253010, 190179210, 190195910 (all 3 codes related to lens reconstruction). On the other hand, persons who had not undergone CS were defined as patients without CS (non-CS cohort).

### Variables

Variable used in this study included age, gender, body mass index (BMI), smoking index, season at admission, length of stay in hospital (LOS), Charlson comorbidity index (CCI) [21, 22], comorbidities at index admission (17 disease as the possible risk factors for glaucoma: [23–26] hypertension, hypotension, ischemic heart disease, heart failure, stroke, diabetes, hyperlipidemia, electrolyte disorders, thyroid dysfunction, systemic lupus erythematosus, dementia, depression, mental disorders, cancer, allergies, peptic ulcer and liver insufficiency/failure), concomitant drugs at index admission (systemic oral drugs for the 17 disease listed above) and glaucoma related measure including glaucoma types, topical glaucoma drugs at index admission (see Table 1 for medical and pharmacy codes). Comorbidities and concomitant drugs were identified based on the medical code and the pharmacy code, respectively. Concomitant drugs used in the day of surgery were excluded. Topical glaucoma treatments included prostaglandin analog (PG), β blocker (BB), carbonic anhydrase inhibitor (CAI), α2 agonist (AA), α1 blocker (AB), αβ blocker (ABB), sympathomimetics, rho-associated coiled kinase inhibitor (ROCKI), fixed-combinations and their generics. Patients were categorized into 6 age groups: ≤39, 40–49, 50–59, 60–69, 70–79, ≥80; two gender groups: male, female; four BMI groups: thin (< 18.5), normal (≥18.5 < 25), fat (≥25), not classified; four season groups: spring (April–June), summer (July–September), autumn (October–December), winter (January–March); 4 CCI groups: low (0), medium (1, 2), high (3, 4), very high (≥5) cases.

**Table 1:** Medical and pharmacy codes used for the study

Therapeutic category	ICD-10 codes	Drug codes^a^	Drug generic name
Hypertension	I10	213, 214, 217	
Hypotension	I95%	216	
Ischemic heart disease	I20%-I25%	212, 214, 217	
Heart failure	I50%	211, 213, 214, 217	
Stroke	I63%	333, 339	
Diabetes	E10%-E14%	396	
Hyperlipidemia	E78%	218	
Electrolyte disorders	E87%	321, 322	
Thyroid dysfunction	E02%, E03%	243	
Systemic lupus erythematosus	M32%	2,456,001, 3,999,005, 3,999,038	Prednisolone, Azathioprine, Hydroxychloroquine
Depression	F251, F31%-F35%	1179	
Dementia	F00%-F03%	1,190,012, 1,190,018, 1,190,019	Donepezil, Memantine, Galantamine,
Mental disorders	F09, F23, F28, F29	117	
Cancer	C00%-C97%	421, 422, 423, 424, 429	
Allergies	D690, D721, H011, H101, H169, H200, H650, H651, H654, H659, J301-J304, L23%, L500, T781, T784	441, 442, 443, 449	
Peptic ulcer	F54, K221, K25%, K26%, K27%, K28%, K51%, K626, K633, K828, K838	232	
Liver insufficiency/failure	K70%-K77%	625, 6399, 3919, 3999	
Glaucoma (NTG/POAG/OAG)	H401	131^b^	
Unspecified glaucoma	H409	131^b^	

*ICD-10* international classification of disease, *NTG* normal tension glaucoma, *OAG* open angle glaucoma, *POAG* primary open angle glaucoma

^a^Classified by the Drug Therapeutic Class Code in Japan

^b^Furthermore, commonly prescribed glaucoma eye drops were narrowed down by the Japanese National Health Insurance drug price listing pharmaceuticals code

### Study outcomes

The primary outcome was the risk factors associated with TLE using patients’ baseline demographic and clinical characteristics at the index admission. The secondary outcomes were the rate of comorbidities, the rate of concomitant use of prescribed systemic oral drugs and the treatment patterns of prescribed topical glaucoma drugs (eye drops) at the index admission in the GS and non-GS cohorts. Furthermore, GS cohort was divided into two cohorts based on with or without CS. The primary and secondary outcomes were also performed.

### Statistical analysis

Descriptive analysis was performed for the basic features of the data as in mean with standard deviation (SD) for continuous variables and frequency (n) and percentage (%) for categorical variables. For outcomes, the difference between the GS and non-GS cohorts were compared according to baseline characteristics. Based on variable types and normality, the Chi-square test (or Fisher’s exact test, if cell expectations were less than 5), the Student’s t-test and the Mann-Whitney U test were used to examine differences.

To identify the factors associated with TLE, logistic regression analysis was used. Potentially significant variables on univariable analysis (*p* < 0.05) were considered a priori for inclusion in a multivariable logistic regression model. Pairwise correlation coefficients were examined between variables that were potentially related before inclusion in our multivariable model to avoid collinearity. Stepwise forward-backward elimination analysis was performed and variables with *p* < 0.0001 were retained in the final multivariable model. In order to validate the final model, the variance inflation factor (VIF) was performed to test for multicollinearity among the predictor variables. A VIF exceeding 10 was regarded as indicating serious multicollinearity, and values greater than 4.0 was considered a cause for concern [27–29]. *P* -values, adjusted odds ratios (ORs) and Wald 95% confidence intervals (CIs) were obtained for the predictor variables. Furthermore, stratified analysis by CS were also performed. All the statistical analyses were performed using the JMP Pro Ver 14.0 (SAS Institute Inc., Cary, NC, USA). *P*-values of < 0.05 were considered statistically significant.

## Results

A total of 29,599 patients met all inclusion criteria for this study, of whom 12,038 (40.7%) patients were in the GS cohort and 17,561 (59.3%) patients were in the non-GS cohorts (Fig. 1). The non-GS cohort was considered to be patients who were hospitalized because of the sudden increase in IOP, however, cured and discharged only by drug treatment. Furthermore, it might have been hospitalized for a glaucoma medical checkup. Among the GS cohort, 2991 (24.8%) patients were in the CS cohort and 9047 (75.2%) patients were in the non-CS cohorts.

The baseline demographics and clinical characteristics of the GS and non-GS cohorts are summarized in Table 2. The mean age was 68 years and 55.6% were male, and most patients had a moderate BMI and a low CCI. In unadjusted comparison, the GS cohort significantly had a higher percentage of patients with age 50–59 or 60–69 (*P* < 0.0001 each); male (*P* < 0.0001); normal BMI (*P* = 0.0313); longer LOS (*P* < 0.0001); 2 comorbidities at the index admission: diabetes (*P* = 0.0101) and allergies (*P* < 0.0001); 11 concomitant drugs at index admission: ischemic heart disease, stroke, diabetes, systemic lupus erythematosus, depression, mental disorders, cancer, allergies, peptic ulcer (*P* < 0.0001 each), hyperlipidemia (*P* = 0,0016) and liver insufficiency/failure (*P* = 0.0294); and a diagnosis of primary open angle glaucoma (POAG) (*P* < 0.0001) than the non-GS cohort, whereas significantly had a lower percentage of patients with age ≤ 39 or ≥ 80 (*P* < 0.0001 each); female (*P* < 0.0001); autumn season admission (*P* = 0.0041); 1 comorbidity at index admission: hypertension (*P* = 0.0009): 2 concomitant drugs at index admission: hypertension and heart failure (*P* < 0.0001 each); and a diagnosis of normal tension glaucoma (NTG) (*P* < 0.0001). Concerning the season of hospital admission, we thought that IOP rises in winter and trabeculectomy would be more frequent, however, medical big data such as the Japanese nationwide database which has more than forty million acute care hospitalized patients registered, did not show seasonal difference.

**Table 2:** Baseline demographic and clinical characteristics of the study cohorts

*Variable*	*Glaucoma surgery* *(N = 12,038)*		*Non-glaucoma surgery* *(N = 17,561)*		
	*n*	*Percentage or* *mean ± SD*	*n*	*Percentage or* *mean ± SD*	*P Value*
Age (years)	12,038	68.5 ± 12.4	17,561	68.8 ± 14.0	< 0.0001*
Category					< 0.0001
≤ 39	305	2.5	719	4.1	< 0.0001
40–49	628	5.2	929	5.3	0.7910
50–59	1488	12.4	1842	10.5	< 0.0001
60–69	3227	26.8	4168	23.7	< 0.0001
70–79	4207	34.9	6069	64.6	0.4943
≥ 80	2183	18.1	3034	21.8	< 0.0001
Gender					
Category					< 0.0001
Male	7032	58.4	9376	53.4	< 0.0001
Female	5006	41.6	8185	46.6	< 0.0001
Body mass index (kg/m^2^)	12,038	22.7 ± 4.3	17,561	22.2 ± 6.2	< 0.0001*
Category					< 0.0001
Thin (< 18.5)	1076	8.9	1565	8.9	0.9504
Normal (≥18.5 < 25)	7867	65.4	11,262	64.1	0.0313
Fat (≥25)	2969	24.7	4220	24.0	0.2144
Not classified	126	1.0	514	2.9	< 0.0001
Smoking index	12,038	91.3 ± 288.5	17,561	89.9 ± 290.6 14.0	0.9622*
Maximum	0		0		
Minimum	5000		4995		
Season					
Category					0.0303
Spring (April–June)	3295	27.4	4659	26.5	0.1093
Summer (July–September)	2504	20.8	3644	20.8	0.9187
Autumn (October–December)	2520	20.9	3922	22.3	0.0041
Winter (January–March)	3719	30.9	5336	30.4	0.3553
Length of stay in hospital	12,038	12.1 ± 6.1	17,561	8.6 ± 6.0	< 0.0001*
Maximum	1		1		
Minimum	99		128		
Charlson comorbidity index					
Category					0.4710
Low (0)	9748	81.0	14,287	81.4	0.4135
Medium (1, 2)	2177	18.1	3100	17.7	0.3457
High (3, 4)	109	0.9	162	0.9	0.9013
Very High (≥5)	4	0.0	12	0.1	0.3085
Comorbidities					
Circulatory system					
Hypertension	852	7.1	1428	8.1	0.0009
Hypotension	2	0.0	5	0.0	0.7820
Ischemic heart disease	292	2.4	412	2.3	0.6694
Heart failure	82	0.7	147	0.8	0.1377
Stroke	80	0.7	118	0.7	1.0000
Metabolic system					
Diabetes	1763	14.6	2386	13.6	0.0101
Hyperlipidemia	385	3.2	575	3.3	0.7166
Electrolyte disorders	134	1.1	232	1.3	0.1204
Thyroid dysfunction	14	0.1	28	0.2	0.3512
Systemic lupus erythematosus	10	0.1	28	0.2	0.0971
Nervous system					
Dementia	18	0.2	43	0.2	0.0894
Depression	56	0.5	88	0.5	0.7339
Mental disorders	1	0.0	0	0.0	0.4067
Immune system					
Cancer	110	0.9	155	0.9	0.8017
Allergies	981	8.1	542	3.1	< 0.0001
Gastrointestinal system					
Peptic ulcer	181	1.5	221	1.3	0.0821
Liver insufficiency/failure	30	0.2	35	0.2	0.3784
Concomitant drug					
Circulatory system					
Hypertension	3416	28.4	5546	31.6	< 0.0001
Hypotension	36	0.3	56	0.3	0.8319
Ischemic heart disease	2125	17.7	2625	14.9	< 0.0001
Heart failure	3656	30.4	5735	32.7	< 0.0001
Stroke	2942	24.4	3053	17.4	< 0.0001
Metabolic system					
Diabetes	588	4.9	635	3.6	< 0.0001
Hyperlipidemia	777	6.5	977	5.6	0.0016
Electrolyte disorders	1233	10.2	1907	10.9	0.0909
Thyroid dysfunction	78	0.6	106	0.6	0.6518
Systemic lupus erythematosus	303	2.5	301	1.7	< 0.0001
Nervous system					
Dementia	45	0.4	84	0.5	0.2084
Depression	2518	20.9	2005	11.4	< 0.0001
Mental disorders	2522	21.0	2012	11.5	< 0.0001
Immune system					
Cancer	9313	77.4	3997	22.8	< 0.0001
Allergies	436	3.6	433	2.5	< 0.0001
Gastrointestinal system					
Peptic ulcer	336	18.5	2450	14.9	< 0.0001
Liver insufficiency/failure	2230	2.8	418	2.4	0.0294
Glaucoma types					
POAG	4404	36.6	5628	32.0	< 0.0001
OAG	4214	35.0	6300	36.0	0.1253
NTG	743	6.2	1331	7.6	< 0.0001
Not classified	2677	22.2	4320	24.5	< 0.0001
Glaucoma drug by class					
PG	2827	23.5	3923	22.3	0.0215
BB	713	5.9	1212	6.9	0.0008
CAI	1086	9.0	1445	8.2	0.0168
ROCKI	694	5.8	928	5.3	0.0771
AA	1640	13.3	2317	13.2	0.7534
AB	131	1.1	141	0.8	0.0130
ABB	14	0.1	17	0.1	0.7151
Sympathomimetics	12	0.1	11	0.1	0.2655
PG/BB fixed combination	296	2.5	425	2.4	0.8479
CAI/BB fixed combination	713	5.9	1212	6.9	0.0008
Glaucoma drug by generic name					
PG					
Isopropyl Unoprostone	4	0.0	13	0.1	0.2165
Isopropyl Unoprostone GE	0	0.0	0	0.0	NA
Latanoprost	917	7.6	1272	7.2	0.2309
Latanoprost GE	152	1.3	238	1.4	0.5050
Travoprost	377	3.1	550	3.1	1.0000
Travoprost GE	0	0.0	0	0.0	NA
Tafluprost	367	3.0	669	3.8	0.0004
Tafluprost GE	0	0.0	0	0.0	NA
Bimatoprost	1069	8.9	1274	7.3	< 0.0001
Bimatoprost GE	0	0.0	0	0.0	NA
BB					
Timolol Maleate	435	3.6	761	4.3	0.0020
Timolol Maleate GE	52	0.4	73	0.4	0,8544
Carteolol Hydrochloride	217	1.8	367	2.1	0.0813
Carteolol Hydrochloride GE	10	0.1	12	0.1	0.6687
Betaxolol Hydrochloride	2	0.0	5	0.0	0.7082
Betaxolol Hydrochloride GE	0	0.0	1	0.0	1.0000
CAI					
Dorzolamide Hydrochloride	225	1.9	295	1.7	0.2243
Dorzolamide Hydrochloride GE	0	0.0	0	0.0	NA
Brinzolamide	686	5.7	914	5.2	0.0670
Brinzolamide GE	0	0.0	0	0.0	NA
ROCKI					
Ripasudil Hydrochloride Hydrate	694	5.8	928	5.3	0.0771
Ripasudil Hydrochloride Hydrate GE	0	0.0	0	0.0	NA
AA					
Brimonidine Tartrate	1604	13.3	2317	13.2	0.7534
Brimonidine Tartrate GE	0	0.0	0	0.0	NA
AB					
Bunazosin Hydrochloride	131	1.1	141	0.8	0.0130
Bunazosin Hydrochloride GE	0	0.0	0	0.0	NA
ABB					
Levobunolol Hydrochloride	2	0.0	1	0.0	0.5705
Levobunolol Hydrochloride GE	0	0.0	0	0.0	NA
Nipradilol	13	0.1	16	0.1	0.7067
Nipradilol GE	0	0.0	1	0.0	1.0000
Sympathomimetics					
Dipivefrin Hydrochloride	12	0.1	11	0.1	0.2917
Dipivefrin Hydrochloride GE	0	0.0	0	0.0	NA
PG/BB fixed combination					
Lat/Tim	114	0.9	211	1.2	0.0409
Lat/Tim GE	0	0.0	0	0.0	NA
Lat/Car	1	0.0	3	0.0	0.6560
Lat/Car GE	0	0.0	0	0.0	NA
Tra/Tim	141	1.2	163	0.9	0.0459
Tra/Tim GE	0	0.0	0	0.0	NA
Taf/Tim	41	0.3	51	0.3	0.4582
Taf/Tim GE	0	0.0	0	0.0	NA
CAI/BB fixed combination					
Dor/Tim	1519	12.6	2327	13.3	0.1133
Dor/Tim GE	0	0.0	0	0.0	NA
Brinzolamide/Tim	359	3.0	547	3.1	0.5364
Brinzolamide/Tim GE	0	0.0	0	0.0	NA

*AA* α2-agonist, *AB* α1-blocker, *ABB* αβ-blocker, *BB* β-blocker, *CAI* carbonic anhydrase inhibitor, *Dor* dorzolamide hydrochloride, *GE* generic, *Lat* latanoprost, *NA* not assessed, *NTG* normal tension glaucoma, *OAG* open angle glaucoma, *PG* prostaglandin analog, *POAG* primary open angle glaucoma, *ROCKI* rho-associated protein kinase inhibitor, *SD* standard deviation, *Taf* tafluprost, *Tim* timolol maleate, *Tra* travoprost

*Calculated using the Mann-Whitney U test; the remaining *P* Values were calculated with the Chi-square test or Fisher’s exact test

Regarding the primary outcome on the risk factors associated with TLE, the 28 variables previously mentioned were included in the initial regression model. Stepwise logistic regression with forward-backward elimination retained 11 of the 20 variables as the significant predictors (*P* < 0.0001). The VIFs for the predictor variables in this study were all < 4.0, indicating the absence of multicollinearity. Table 3 lists the variables estimated for the final model. The use of cancer drug (adjusted OR: 0.0862, 95% CI: 0.0816–0.0911) and having allergies including systemic and topical (adjusted OR: 0.3590, 95% CI: 0.3223–0.3997) were the most significant predictors of TLE, followed by using concomitant drugs including depression, ischemic heart disease and peptic ulcer; being diagnosed with POAG; and longer LOS. In contrast, the use of hypertension drug (adjusted OR: 1.1651, 95% CI: 1.1073–1.2258) and having hypertension (adjusted OR: 1.1621, 95% CI: 1.0640–1.2693) was most strongly associated with reduced likelihood of TLE followed by age ≥ 80 and female. When we restricted the multivariable analysis to the subgroup of patients with TLE who had undergone CS, the association between increase CS and baseline characteristics (*P* < 0.0001); age 70–79 and being diagnosed with POAG were also significant in the model (Table 4).

**Table 3:** Logistic regression analysis of factors associated with or without trabeculectomy surgery

*Variable*	*Estimate*	*Adjusted OR (95% CI)*	*P Value*
Hypertension drug	0.649155	1.1651(1.1073–1.2258)	< 0.0001
Hypertension	0.33362	1.1621(1.0640–1.2693)	< 0.0001
Age ≥ 80	0.174507	1.2609(1.0189–1.3370)	< 0.0001
Female	0.151442	1.2263(1.1702–1.2851)	< 0.0001
Length of stay in hospital	−0.06979	0.8942 (0.8899–0.8984)	< 0.0001
POAG	−0.17228	0.8175(0.7789–0.8584)	< 0.0001
Peptic ulcer drug	−0.27303	0.7131(0.6697–0.7593)	< 0.0001
Ischemic heart disease drug	−0.29949	0.8199(0.7701–0.8728)	< 0.0001
Depression drug	−0.4121	0.4873(0.4571–0.5195)	< 0.0001
Allergies	−0.57812	0.3590(0.3223–0.3997)	< 0.0001
Cancer drug	−2.23632	0.0862(0.0816–0.0911)	< 0.0001

*CI* confidence interval, *OR* odd ratio, *POAG* primary open angle glaucoma

**Table 4:** Logistic regression analysis of factors associated with or without combined trabeculectomy and cataract surgery

*Variable*	*Estimate*	*Adjusted OR (95% CI)*	*P Value*
Age ≤ 39	2.62523	1.2066(6.2634–25.5751)	< 0.0001
Age 40–49	1.603633	47,396(3.4675–6.4783)	< 0.0001
Age 50–59	0.965275	2.6394(2.2454–3.1026)	< 0.0001
Cancer drug	0.384652	1.4361(1.3062–1.5789)	< 0.0001
Hyperlipidemia drug	0.194787	1.2737(1.0657–1.5222)	< 0.0001
POAG	−0.17353	0.8450(0.7761–0.9199)	< 0.0001
Age 70–79	−0.28579	0.5642(0.5184–0.6140)	< 0.0001

*CI* confidence interval, *OR* odd ratio, *POAG* primary open angle glaucoma

**Fig. 2:**
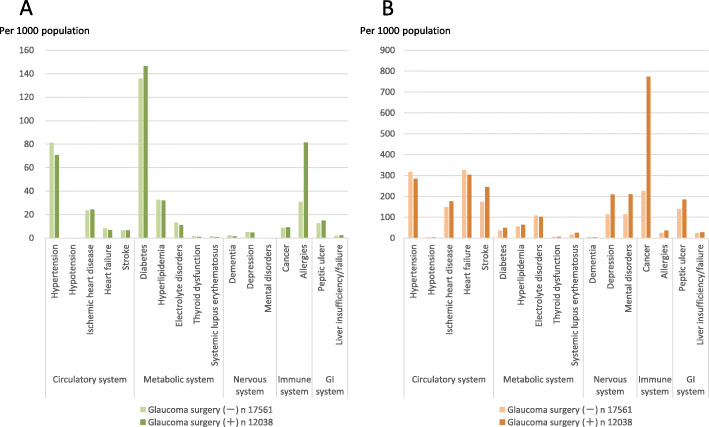
**a** Prevalence rate of comorbidities, **b** rate of concomitant use of prescribed systemic oral drugs in the glaucoma surgery and non-glaucoma surgery cohorts

The rate of comorbidities and the rate of concomitant use of prescribed systemic oral drugs in the GS and non-GS cohorts are shown in Fig. 2. The most common comorbidity was diabetes in both, the GS (14.6%) and non-GS cohort (13.6%). Diabetes was followed by allergy, hypertension, hyperlipidemia and Ischemic heart disease. Allergy was remarkably higher in the GS cohort (Fig. 2a). Concomitant drugs use with frequency > 10% and significant difference in the two cohorts were hypertension, Ischemic heart disease, heart failure, stroke, depression, mental disorders, cancer and peptic ulcer. The use of cancer drug was remarkably higher in the GS cohort. (Fig. 2b). On the other hand, mitomycin C (MMC) originally was used as a systemic chemotherapeutic agent, and it has been used widely in ophthalmic practice, during and after surgery, for enhancing the success rate of glaucoma filtration surgery. Since concomitant drugs were limited to oral drugs and were excluded drugs used in the day of surgery, this study did not include MMC in the cancer drugs.

**Fig. 3:**
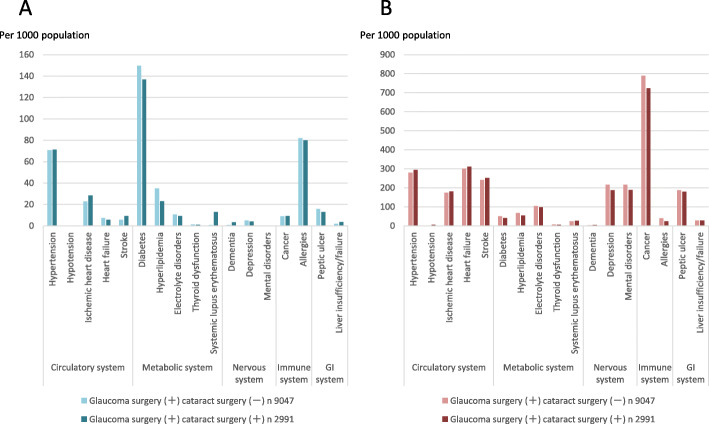
**a** Prevalence rate of comorbidities, **b** rate of concomitant use of prescribed systemic oral drugs in the cataract surgery and non-cataract surgery combined with glaucoma surgery cohorts

Among the GS cohort, the rate of comorbidities and the rate of concomitant use of prescribed systemic oral drugs in the CS and non-CS cohorts are shown in Fig. 3. The most common comorbidity was diabetes in both, the CS (17.7%) and non-CS cohort (19.3%). Diabetes was followed by allergy and hypertension; however, the lack of significant differences was shown between the two cohorts (Fig. 3a). Concomitant drugs use with frequency > 10% and significant difference in the two cohorts were depression, mental disorders, and cancer (Fig. 3b).

**Fig. 4:**
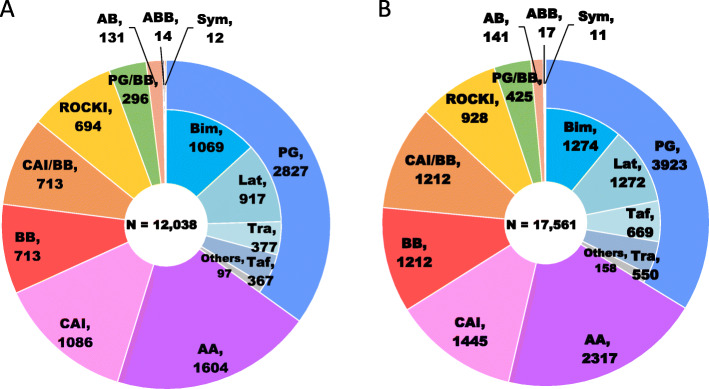
Treatment patterns of prescribed topical glaucoma drugs (eye drops) at the index admission in the glaucoma surgery (**a**) and non-glaucoma surgery (**b**) cohorts. AA, α2-agonist; AB. α1-blocker; ABB, αβ-blocker; BB, β-blocker; Bim, bimatoprost; CAI, carbonic anhydrase inhibitor; Lat, latanoprost; PG, prostaglandin analog; ROCKI, Rho-associated protein kinase inhibitor; Sym, sympathomimetics; Taf, tafluprost; Tra, travoprost

**Fig. 5:**
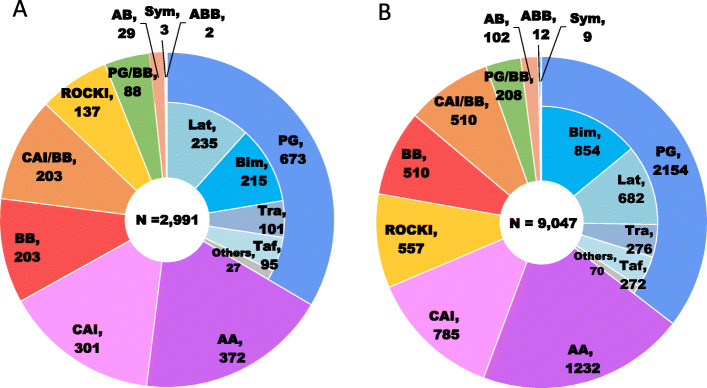
Treatment patterns of prescribed topical glaucoma drugs (eye drops) at the index admission in the cataract surgery (**a**) and non-cataract surgery (**b**) combined with glaucoma surgery cohorts. AA, α2-agonist; AB. α1-blocker; ABB, αβ-blocker; BB, β-blocker; Bim, bimatoprost; CAI, carbonic anhydrase inhibitor; Lat, latanoprost; PG, prostaglandin analog; ROCKI, Rho-associated protein kinase inhibitor; Sym, sympathomimetics; Taf, tafluprost; Tra, travoprost

The treatment patterns of prescribed topical glaucoma drugs (eye drops) at the index admission are summarized in Figs. 4 and 5. In the GS cohort, PG accounted for one third of total glaucoma eye drops. PG [2827 (23.5%)] was followed by AA [1640 (13.3%)], CAI [1086 (9.0%)] and BB [713 (5.9%)] by drug class; the most commonly used as first-line treatment was bimatoprost (8.9%) followed by latanoprost (7.6%), travoprost (3.1%) and tafluprost (3.0%) by generic name, including only original drugs not generic drugs (Fig. 4a). In the non-GS cohort, which has the similar treatment pattern to the GS cohort, PG [3923 (22.3%)], AA [2317 (13.2%)], CAI [1445 (8.2%)] and BB [1212 (6.9%)] were used by drug class, and the most commonly used as first-line treatment was bimatoprost (7.3%), followed by latanoprost (7.2%), tafluprost (3.8%) and travoprost (3.1%) by generic name (Fig. 4b). In the CS cohort, PG accounted for one third of total glaucoma eye drops. PG [673 (29.2%)] was followed by AA [372 (16.2%)], CAI [301 (13.1%)] and BB [203 (8.8%)] by drug class; the most commonly used as first-line treatment was latanoprost (10.2%), followed by bimatoprost (9.3%), travoprost (4.4%) and tafluprost (4.1%) by generic name (Fig. 5a). In the non-CS cohort, which has the similar treatment pattern to the CS cohort, PG [2154 (30.7%)], AA [1232 (17.6%)], CAI [785 (11.2%)] and ROCK [557 (7.9%)] were used by drug class, and the most commonly used as first-line treatment was bimatoprost (12.2%), followed by latanoprost (9.7%), travoprost (3.9%) and tafluprost (3.9%) by generic name (Fig. 5b).

## Discussion

To date, we found no other studies that identified the risk factors associates with TLE in patients with glaucoma in Japan or other countries. Our study did and revealed a significant influence of 11variables on TLE extracted from the claims database. Of these, 7 were significant more likely to increase TLS, and 4 were significant more likely to decrease TLE. The immune-related comorbidities and concomitant drugs use were the most likely to be TLE. Although there was no difference in the treatment pattern of prescribed glaucoma eye drops between the GS cohort and the non-GS cohort, the rate of the comorbidities and the rate of concomitant drugs use were similar trend to the above identified variables. Therefore, careful management of glaucoma patients with these variables may be important factor for reducing glaucoma surgery burden.

In Japan, TLO is also often preferred as glaucoma surgery, and TLE may target relatively severe glaucoma patients. Since this study used DPC data collected form patients who were admitted to the nationwide acute care hospitals, we assumed that patients with relatively severe glaucoma were included and considered appropriate to select only TLE among GS. On the other hand, since TLO such as microhook TLO is often additionally used during CS, it is difficult to be an indicator for estimating exacerbation risk of glaucoma.

Among the 17 disease as the possible risk factors for glaucoma [23–26] (see *Variable* in the Method section), allergies were identified as the risk factors of comorbidities leading to TLE. Our finding might be explained by the previous studies: the toll-like receptor 4 (TLR4), a transmembrane receptor that mediates immune responses to exogenous, is associated with the risk of NTG [30]. The microglia are related to the immunocompetent cells of the central nervous system, and microglial activation has been reported in glaucoma [31], which might contribute to a higher prevalence of immune-related comorbidities such as allergies. Use of steroids by any route, however, can lead to increased IOP and can cause optic neuropathy resulting in steroid-induced glaucoma [32, 33]. Although our study included oral prednisolone use during the admissions (approximately 2.0%), a history of corticosteroids use was excluded. Also, steroid-induced glaucoma (ICD-10: H406) was excluded so that corticosteroids as a potential confounding factor would be less influence between allergies and glaucoma.

Among systemic oral drugs for the 17 disease, cancer, depression, ischemic heart disease or peptic ulcer were identified as the risk factors of concomitant drugs leading to GS. According to the previous studies, the downregulation of cell cycle progression by checkpoint inhibitors has recently been targeting for cancer therapy [34, 35], can cause cell death beyond cancer cells, and therefore may induce neurodegenerative disorders such as glaucoma [36]. However, one of the risk factors for glaucoma is increased oxidative stress, and drugs targeting oxidative stress in cancer could reduce the oxidative stress-induced apoptosis of retinal ganglion cells in glaucoma [37, 38]. Therefore, we have not been able to identify reports from previous studies of whether cancer drugs is associated with TLE. According to Table 2, only 110 patients in the GS cohort (0.9%) and 155 patients in the non-GS cohort (0.9%) had cancer. However, 9313 patients in the GS cohort (77.4%) and 3997 patients in the non-GS cohort (22.8%) used medications for cancer. From these, the GS cohort used more medication for cancer than the non-GS cohort and would be considered more sever stage of glaucoma. Since pharmacy codes that used medications for cancer include 421 (alkylating agents), 422 (antimetabolites), 423 (antibiotics), and 424 (plant extract preparations), cancer treatments may have been used not only for cancer patients but also non-cancer patients.

Other studies report that depression is strongly linked with glaucoma [39] and results in elevated oxidative stress [40]. Likewise, ischemic heart disease is linked with glaucoma probably affect vasculature dysfunction [41]. Furthermore, many factors contribute to peptic ulcer including glaucoma [42] is another likely reason that may contribute to the positive association. In contrast, hypertension and its drug were identified as the risk factors leading to non-GS. Our finding might to be in line with the previous studies that hypertension improve ocular blood flow [43], however, hypertension oral administration is a risk of glaucoma progression [44]. Stratified analysis based in CS showed that none of the 17 disease were identified as the risk factors leading to CS combined with TLE.

In addition, patients with POAG was also identified as the significant more likely to be TLE. The rate for glaucoma type in subjects 40 years of age and older was estimated at 5.0% for all glaucoma, 0.3% for POAG, and 3.6% for NTG [5]. In contrast, this study showed that the rates of POAG, OAG and NTG at the index admission were 36.6, 35.0 and 6.2%, respectively, in the GS cohort, and 32.0, 36.0 and 7.6%, respectively, in the non-GS cohort. The ratios of NTG seem to be fewer than expected. One possible explanation could be that the rate of progress of glaucomatous optic neuropathy among patients with NTG is generally slower than that among those with other types of glaucoma, therefore, patients with NTG have less need to go to DPC hospitals.

Furthermore, this study identified longer LOS as the factors associate with TLE. Japanese hospitals generally provide rehabilitation and nursing care in addition to acute medical care, which may contribute to the longer LOS.

Patients in both cohorts, the high rate of comorbidities and concomitant drugs use were similar trend to the above identified variables. In the GS cohort, allergy and cancer drugs were most significantly higher than the non-GS cohort. Diabetes was the most common comorbidities in both, the GS (14.6%) and non-GS cohort (13.6%), and significantly higher in the GS cohorts, but not identified as the risk factors leadings to GS. On the other hand, the rate of the diabetes drug use was low in both, the GS (4.9%) and non-GS cohort (3.6%). Since the diabetes drug has been reported as both a risk factor and a protective factor for glaucoma [23–26], this study show that few patients with glaucoma who had diabetes may be treated with the diabetes drug.

The treatment patterns of prescribed glaucoma eye drops at the index admission was similar between the two cohorts; PG was most commonly prescribed, AA and CAI were second and third, respectively. Our result is in line with the data from a published report indicating that the most commonly used first-line monotherapy was a PG [45–48], while CAI/BB was the most commonly used fixed combination as first- and second-line treatment [49, 50]. Although BB is also recommended as a first-line monotherapy in the guideline for glaucoma, the prescription rate of BB was low in this study. This is probably because the patients had comorbidities of asthmas, chronic obstructive pulmonary disease or heart failure may not prescribed BB according to the respective drug information. Or elderly patients may have difficult using BB. On the other hand, BB was considered to be common in the GS cohorts because of bradycardia, but not so in our results. The ROCKI as well as the EP2 receptor agonist become available in Japan recently and has been reported to show an additional IOP-lowering in combination with other glaucoma ophthalmic solutions [51–54]. Thus, the prescription trend for glaucoma eye drops may change in the future.

This study had several limitations. First, we included only DPC hospitals with glaucoma beds, so the results may not be generalizable; however, DPC database contains detailed medical data on numerous patients residing throughout the Japan in a broad array of geographic regions. Moreover, the variables included in the final predictive model are available in other Japanese administrative claims databases. Second, limitations common to studies using administrative claims data apply to this study [55–58]. These limitations include lack of certain information in the database and errors or omissions in claims coding. Third, claims data lack clinical information (such as IOP, visual field, etc.) to access disease severity. Therefore, it was not possible to evaluate whether the severity level of documented comorbid conditions was comparable between our study cohorts and whether different stages of glaucoma were associated with specific comorbidity profiles. Fourth, our data did not exclude laser trabeculoplasty (LT). Although, LT reported to be an alternative to topical glaucoma drug treatment and the same IOP-lowering effect as eye drops as monotherapy [59], it may have influenced the results of our analysis. Fifth, unmeasured confounders may limit the findings. Finally, the present analyses were built according to the assumption that all the claimed drugs were used by the patients. To address these limitations, we need to conduct further studies using the real-world data combined with clinical data.

## Conclusions

The results of this study show that the risk factors leading to TLE of glaucoma patient can be predicted with 7 commonly available demographic and administrative claim-based variables including: having comorbidities related to allergies; taking concomitant drugs related to cancer, depression, ischemic heart disease and peptic ulcer; being diagnosed with POAG, and longer LOS. Furthermore, the rate of comorbidities and the rate of concomitant drug use were similar tendency as the above identified variables. Therefore, before starting glaucoma treatment, special focus on Japanese patients with glaucoma, especially POAG, who have allergy-related comorbidities, or take the immune, nervous, circulatory or gastrointestinal system-related concomitant drugs, through medical interview by ophthalmologists seems to be desirable.

## Supplementary Information

**Additional file 1: Supplemental Table 1**. Baseline demographic and clinical characteristics of the study subgroups.

## Data Availability

The data that support the findings of this study are available from the DPC Study Group, a government-funded academic group, but restrictions apply to the availability of these data, which were used under license for the current study. Therefore, the data are not publicly available. The data are however available from the authors upon reasonable request and with the permission of the DPC Study Group.

## Abbreviations

AA: α2 agonist
AB: α1 blocker
ABB: αβ blocker
BB: β blocker
BMI: Body mass index
CAI: Carbonic anhydrase inhibitor
CCI: Charlson comorbidity index
CIs: Confidence intervals
CS: Cataract surgery
DPC: Diagnosis procedure combination
GS: Glaucoma surgery
ICD: International statistical classification of disease
IOP: Intraocular pressure
LOS: Length of stay in hospital
LT: Laser trabeculoplasty
NTG: Normal tension glaucoma
OAG: Open angle glaucoma
OR: Odds ratio
PG: Prostaglandin analog
POAG: Primary open angle glaucoma
ROCKI: Rho-associated coiled kinase inhibitor
SD: Standard deviation
TLO: Trabeculotomy
TLE: Trabeculectomy
VIF: Variance inflation factor
