# Supplementary material for: Risk factors leading to trabeculectomy surgery of glaucoma patient using Japanese nationwide administrative claims data: a retrospective non-interventional cohort study
Source: BMC Ophthalmol. 2021 Mar 29;21:153. doi: 10.1186/s12886-021-01897-4 (PMC8008563; doi:10.1186/s12886-021-01897-4)
Supplement: Supplementary file 1 — Additional file 1: Supplemental Table 1. Baseline demographic and clinical characteristics of the study subgroups. [file 12886_2021_1897_MOESM1_ESM.docx]

Supplemental Table 1. Baseline demographic and clinical characteristics of the study subgroups

|  | *Glaucoma surgery* | | | | |  |  |
| --- | --- | --- | --- | --- | --- | --- | --- |
| *Variable* | *Cataract surgery (N =2,991)* | |  | *Non-cataract  (N = 9,047)* | |  |  |
|  | *n* | *Percentage or*  *mean ± SD* |  | *n* | *Percentage or*  *mean ± SD* |  | *P Value* |
| Age (years) | 2991 | 72.0 ±8.9 |  | 9047 | 67.4 ±13.2 |  | <0.0001*** |
| Category | 1234567 |  |  | 1234567 |  |  | <0.0001 |
| ≤39 | 8 | 0.3 |  | 297 | 3.3 |  | <0.0001 |
| 40-49 | 43 | 1.4 |  | 585 | 6.5 |  | <0.0001 |
| 50-59 | 180 | 6.0 |  | 1308 | 14.5 |  | <0.0001 |
| 60-69 | 834 | 27.9 |  | 2393 | 26.5 |  | 0.1276 |
| 70-79 | 1347 | 45.0 |  | 2860 | 31.6 |  | <0.0001 |
| ≥80 | 579 | 19.4 |  | 1640 | 17.7 |  | 0.0457 |
| Gender |  |  |  |  |  |  |  |
| Category |  |  |  |  |  |  | 0.2568 |
| Male | 1774 | 59.3 |  | 5258 | 58.1 |  | 0.2568 |
| Female | 1217 | 40.7 |  | 3789 | 41.9 |  | 0.2568 |
| Body mass index (kg/m^2^) | 2991 | 22.7 ±4.3 |  | 9047 | 22.8 ±4.3 |  | 0.7096*** |
| Category |  |  |  |  |  |  | 0.9071 |
| Thin (<18.5) | 272 | 9.1 |  | 804 | 8.9 |  | 0.7394 |
| Normal (≥18.5 <25) | 1939 | 64.8 |  | 5928 | 65.5 |  | 0.4921 |
| Fat (≥25) | 747 | 25.0 |  | 2222 | 24.6 |  | 0.6597 |
| Not classified | 33 | 1.1 |  | 93 | 1.0 |  | 0.7559 |
| Smoking index | 2991 | 96.5 ±309.6 |  | 9407 | 89.5 ±281.2 |  | 0.0005*** |
| Maximum | 0 |  |  | 0 |  |  |  |
| Minimum | 5000 |  |  | 4000 |  |  |  |
| Season |  |  |  |  |  |  |  |
| Category |  |  |  |  |  |  | 0.0081 |
| Spring (April- June) | 840 | 28.1 |  | 2455 | 27.1 |  | 0.3205 |
| Summer (July-September) | 668 | 22.2 |  | 1839 | 20.3 |  | 0.0272 |
| Autumn (October-December) | 632 | 21.1 |  | 1888 | 20.9 |  | 0.7558 |
| Winter (January-March) | 854 | 28.6 |  | 2865 | 31.7 |  | 0.0014 |
| Length of stay in hospital | 2991 | 11.9 ±5.8 |  | 9047 | 12.1 ±6.1 |  | 0.1250*** |
| Maximum | 1 |  |  | 1 |  |  |  |
| Minimum | 77 |  |  | 99 |  |  |  |
| Charlson comorbidity index |  |  |  |  |  |  |  |
| Category |  |  |  |  |  |  | 0.3638 |
| Low (0) | 2425 | 81.1 |  | 7323 | 80.9 |  | 0.8931 |
| Medium (1-2) | 545 | 18.2 |  | 1632 | 18.0 |  | 0.8265 |
| High (3-4) | 21 | 0.7 |  | 88 | 1.0 |  | 0.2200 |
| Very High (≥5) | 0 | 0.0 |  | 4 | 0.0 |  | 0.5781 |
| Comorbidities |  |  |  |  |  |  |  |
| Circulatory system |  |  |  |  |  |  |  |
| Hypertension | 213 | 9.2 |  | 639 | 9.1 |  | 0.9019 |
| Hypotension | 0 | 0.0 |  | 2 | 0.0 |  | 1.0000 |
| Ischemic heart disease | 85 | 3.7 |  | 207 | 3.0 |  | 0.0996 |
| Heart failure | 17 | 0.7 |  | 65 | 0.9 |  | 0.4428 |
| Stroke | 28 | 1.2 |  | 52 | 0.7 |  | 0,0382 |
| Metabolic system |  |  |  |  |  |  |  |
| Diabetes | 409 | 17.7 |  | 1354 | 19.3 |  | 0.0837 |
| Hyperlipidemia | 69 | 3.0 |  | 316 | 4.5 |  | 0.0012 |
| Electrolyte disorders | 28 | 1.2 |  | 95 | 1,4 |  | 0.2685 |
| Thyroid dysfunction | 3 | 0.1 |  | 11 | 0.2 |  | 1.0000 |
| Systemic lupus erythematosus | 39 | 1.7 |  | 7 | 0.1 |  | 0.7172 |
| Nervous system |  |  |  |  |  |  |  |
| Dementia | 10 | 0.4 |  | 8 | 0.1 |  | 0.0051 |
| Depression | 12 | 0.5 |  | 44 | 0.6 |  | 0.6433 |
| Mental disorders | 0 | 0.0 |  | 1 | 0.0 |  | 1.0000 |
| Immune system |  |  |  |  |  |  |  |
| Cancer | 28 | 1.2 |  | 82 | 1.2 |  | 0.9117 |
| Allergies | 239 | 10.4 |  | 742 | 10.6 |  | 0.7288 |
| Gastrointestinal system |  |  |  |  |  |  |  |
| Peptic ulcer | 39 | 1.7 |  | 142 | 2.0 |  | 0.3404 |
| Liver insufficiency/failure | 11 | 0.5 |  | 19 | 0.3 |  | 0.1406 |
| Concomitant drug |  |  |  |  |  |  |  |
| Circulatory system |  |  |  |  |  |  |  |
| Hypertension | 881 | 38.2 |  | 2535 | 36.1 |  | 0.1344 |
| Hypotension | 16 | 0.7 |  | 20 | 0.3 |  | 0.0107 |
| Ischemic heart disease | 540 | 23.4 |  | 1585 | 22.6 |  | 0.5069 |
| Heart failure | 932 | 40.4 |  | 2724 | 38.8 |  | 0.2811 |
| Stroke | 754 | 32.7 |  | 2188 | 31.2 |  | 0.2590 |
| Metabolic system |  |  |  |  |  |  |  |
| Diabetes | 124 | 5.4 |  | 464 | 6.6 |  | 0.0313 |
| Hyperlipidemia | 162 | 7.0 |  | 615 | 8.8 |  | 0.0077 |
| Electrolyte disorders | 291 | 12.6 |  | 942 | 13.4 |  | 0.2968 |
| Thyroid dysfunction | 19 | 0.8 |  | 59 | 0.8 |  | 1.0000 |
| Systemic lupus erythematosus | 81 | 3.5 |  | 222 | 3.2 |  | 0.4589 |
| Nervous system |  |  |  |  |  |  |  |
| Dementia | 14 | 0.6 |  | 31 | 0.4 |  | 0.3865 |
| Depression | 562 | 24.4 |  | 1956 | 27.9 |  | 0.0009 |
| Mental disorders | 564 | 24.4 |  | 1958 | 27.9 |  | 0.0012 |
| Immune system |  |  |  |  |  |  |  |
| Cancer | 2165 | 93.8 |  | 7148 | 101.9 |  | <0.0001 |
| Allergies | 72 | 3.1 |  | 364 | 5.2 |  | <0.0001 |
| Gastrointestinal system |  |  |  |  |  |  |  |
| Peptic ulcer | 84 | 3.6 |  | 1692 | 24.1 |  | 0.4001 |
| Liver insufficiency/failure | 538 | 23.3 |  | 252 | 3.6 |  | 0.9490 |
| Glaucoma types |  |  |  |  |  |  |  |
| POAG | 1183 | 39.6 |  | 3221 | 35.6 |  | 0.0001 |
| OAG | 989 | 33.1 |  | 3225 | 35.6 |  | 0.0103 |
| NTG | 203 | 6.8 |  | 540 | 6.0 |  | 0.1145 |
| Not classified | 616 | 20.6 |  | 2061 | 22.8 |  | 0.0129 |
| Glaucoma drug by class |  |  |  |  |  |  |  |
| PG | 673 | 29.2 |  | 2154 | 30.7 |  | 0.1490 |
| BB | 203 | 8.8 |  | 510 | 7.3 |  | 0.0227 |
| CAI | 301 | 13.1 |  | 785 | 11.2 |  | 0.0225 |
| ROCKI | 137 | 5.9 |  | 557 | 7.9 |  | 0.0011 |
| AA | 372 | 16.2 |  | 1232 | 17.6 |  | 0.1001 |
| AB | 29 | 1.3 |  | 102 | 1.5 |  | 0.5420 |
| ABB | 2 | 0.1 |  | 12 | 0.2 |  | 0.5393 |
| Sympathomimetics | 3 | 0.1 |  | 9 | 9 |  | 1.0000 |
| PG/BB fixed combination | 88 | 3.8 |  | 208 | 3.0 |  | 0.0563 |
| CAI/BB fixed combination | 203 | 8.8 |  | 510 | 7.3 |  | 0.0227 |
| Glaucoma drug by generic name |  |  |  |  |  |  |  |
| PG |  |  |  |  |  |  |  |
| Isopropyl Unoprostone | 0 | 0.0 |  | 4 | 0.1 |  | 0.5781 |
| Isopropyl Unoprostone GE | 0 | 0.0 |  | 0 | 0.0 |  | NA |
| Latanoprost | 235 | 10.2 |  | 682 | 9.7 |  | 0.5778 |
| Latanoprost GE | 43 | 1.9 |  | 109 | 1.6 |  | 0.3446 |
| Travoprost | 101 | 4.4 |  | 276 | 3.9 |  | 0.3645 |
| Travoprost GE | 0 | 0.0 |  | 0 | 0.0 |  | NA |
| Tafluprost | 95 | 4.1 |  | 272 | 3.9 |  | 0.6241 |
| Tafluprost GE | 0 | 0.0 |  | 0 | 0.0 |  | NA |
| Bimatoprost | 215 | 9.3 |  | 854 | 12.2 |  | 0.0002 |
| Bimatoprost GE | 0 | 0.0 |  | 0 | 0.0 |  | NA |
| BB |  |  |  |  |  |  |  |
| Timolol Maleate | 123 | 5.3 |  | 312 | 4.4 |  | 0.1011 |
| Timolol Maleate GE | 20 | 0.9 |  | 32 | 0.5 |  | 0.0348 |
| Carteolol Hydrochloride | 56 | 2.4 |  | 161 | 2.3 |  | 0.7513 |
| Carteolol Hydrochloride GE | 4 | 0.2 |  | 6 | 0.1 |  | 0.2779 |
| Betaxolol Hydrochloride | 1 | 0.0 |  | 1 | 0.0 |  | 0.4352 |
| Betaxolol Hydrochloride GE | 0 | 0.0 |  | 0 | 0.0 |  | NA |
| CAI |  |  |  |  |  |  |  |
| Dorzolamide Hydrochloride | 61 | 2.6 |  | 164 | 2.3 |  | 0.4362 |
| Dorzolamide Hydrochloride GE | 0 | 0.0 |  | 0 | 0.0 |  | NA |
| Brinzolamide | 189 | 8.2 |  | 497 | 7.1 |  | 0.0924 |
| Brinzolamide GE | 0 | 0.0 |  | 0 | 0.0 |  | NA |
| ROCKI |  |  |  |  |  |  |  |
| Ripasudil Hydrochloride Hydrate | 137 | 5.9 |  | 557 | 7.9 |  | 0.0011 |
| Ripasudil Hydrochloride Hydrate GE | 0 | 0.0 |  | 0 | 0.0 |  | NA |
| AA |  |  |  |  |  |  |  |
| Brimonidine Tartrate | 372 | 16.2 |  | 1232 | 17.6 |  | 0.1001 |
| Brimonidine Tartrate GE | 0 | 0.0 |  | 0 | 0.0 |  | NA |
| AB |  |  |  |  |  |  |  |
| Bunazosin Hydrochloride | 29 | 1.3 |  | 102 | 1.5 |  | 0.5420 |
| Bunazosin Hydrochloride GE | 0 | 0.0 |  | 0 | 0.0 |  | NA |
| ABB |  |  |  |  |  |  |  |
| Levobunolol Hydrochloride | 1 | 0.0 |  | 1 | 0.0 |  | 0.4352 |
| Levobunolol Hydrochloride GE | 0 | 0.0 |  | 0 | 0.0 |  | NA |
| Nipradilol | 2 | 0.1 |  | 11 | 0.2 |  | 0.5392 |
| Nipradilol GE | 0 | 0.0 |  | 0 | 0.0 |  | NA |
| Sympathomimetics |  |  |  |  |  |  |  |
| Dipivefrin Hydrochloride | 3 | 0.1 |  | 9 | 0.1 |  | 1.0000 |
| Dipivefrin Hydrochloride GE | 0 | 0.0 |  | 0 | 0.0 |  | NA |
| PG/BB fixed combination |  |  |  |  |  |  |  |
| Lat/Tim | 31 | 1.3 |  | 83 | 1.2 |  | 0.5860 |
| Lat/Tim GE | 0 | 0.0 |  | 0 | 0.0 |  | NA |
| Lat/Car | 1 | 0.0 |  | 0 | 0.0 |  | 0.2485 |
| Lat/Car GE | 0 | 0.0 |  | 0 | 0.0 |  | NA |
| Tra/Tim | 40 | 1.7 |  | 101 | 1.4 |  | 0.3278 |
| Tra/Tim GE | 0 | 0.0 |  | 0 | 0.0 |  | NA |
| Taf/Tim | 16 | 0.7 |  | 25 | 0.4 |  | 0.0451 |
| Taf/Tim GE | 0 | 0.0 |  | 0 | 0.0 |  | NA |
| CAI/BB fixed combination |  |  |  |  |  |  |  |
| Dor/Tim | 341 | 14.8 |  | 1178 | 16.8 |  | 0.0207 |
| Dor/Tim GE | 0 | 0.0 |  | 0 | 0.0 |  | NA |
| Brinzolamide/Tim | 76 | 3.3 |  | 283 | 4.0 |  | 0.1070 |
| Brinzolamide/Tim GE | 0 | 0.0 |  | 0 | 0.0 |  | NA |

AA, α2-agonist; AB, α1-blocker; ABB, αβ-blocker; BB, β-blocker; CAI, carbonic anhydrase inhibitor; Dor, dorzolamide hydrochloride ; GE, generic; Lat, latanoprost; NA, not assessed; NTG, normal tension glaucoma; OAG, open angle glaucoma; PG, prostaglandin analog; POAG, primary open angle glaucoma; ROCKI, rho-associated protein kinase inhibitor; SD, standard deviation; Taf, tafluprost; Tim, timolol maleate; Tra, travoprost

* Calculated using the Mann-Whitney U test; the remaining P Values were calculated with the Chi-square test or Fisher's exact test
